# Structural basis for the molecular recognition of polyadenosine RNA by Nab2 Zn fingers

**DOI:** 10.1093/nar/gkt876

**Published:** 2013-09-25

**Authors:** Sonja I. Kuhlmann, Eugene Valkov, Murray Stewart

**Affiliations:** MRC Laboratory of Molecular Biology, Francis Crick Avenue, Cambridge Biomedical Campus, Cambridge CB2 0QH, UK

## Abstract

The yeast poly(A) RNA binding protein, Nab2, facilitates poly(A) tail length regulation together with targeting transcripts to nuclear pores and their export to the cytoplasm. Nab2 binds polyadenosine RNA primarily through a tandem repeat of CCCH Zn fingers. We report here the 2.15 Å resolution crystal structure of Zn fingers 3–5 of *Chaetomium thermophilum* Nab2 bound to polyadenosine RNA and establish the structural basis for the molecular recognition of adenosine ribonucleotides. Zn fingers 3 and 5 each bind two adenines, whereas finger 4 binds only one. In each case, the purine ring binds in a surface groove, where it stacks against an aromatic side chain, with specificity being provided by a novel pattern of H-bonds, most commonly between purine N6 and a Zn-coordinated cysteine supplemented by H-bonds between purine N7 and backbone amides. Residues critical for adenine binding are conserved between species and provide a code that allows prediction of finger-binding stoichiometry based on their sequence. Moreover, these results indicate that, in addition to poly(A) tails, Nab2 can also recognize sequence motifs elsewhere in transcripts in which adenosines are placed at key positions, consistent with its function in mRNP organization and compaction as well as poly(A) tail length regulation.

## INTRODUCTION

Before the nuclear phase of the gene expression pathway is completed by the export of mRNA to the cytoplasm through nuclear pore complexes (NPCs), nascent transcripts progress through a co-ordinated series of modifications, including 5′-capping, splicing and 3′-cleavage/polyadenylation, that are mediated by a host of mRNA-binding proteins (1–7). Moreover, this process is also monitored by a complex surveillance apparatus that prevents the export of incorrectly processed transcripts (8). In *Saccharomyces cerevisiae*, nuclear export of bulk mRNA is mediated primarily by the export factor Mex67:Mtr2 that binds both mRNPs and NPC proteins (1–8) and facilitates the passage of mature transcripts through the pores. The essential heterogeneous nuclear ribonuclearprotein, Nab2 (nuclear abundant poly(A) RNA binding protein 2), a conserved polyadenosine RNA-binding Zn finger protein, functions in polyadenylation, surveillance and the generation of export-competent mRNPs (9–17). Thus, *nab2* mutants frequently generate hyperadenylation, defects in surveillance and reduced mRNA nuclear export resulting in nuclear accumulation of poly(A)-mRNA (11,16,18–21). However, the severity of these effects varies between mutants, indicating that the different *nab2* phenotypes are separable and result from this protein functioning at several different steps in the gene expression pathway. For example, at the restrictive temperature (14°C), the cold-sensitive *nab2-21* mutant (in which residues 424–445 are deleted) shows both hyperadenylation and mRNA export defects, whereas at the permissive temperature (30°C), only hyperadenylation is seen, consistent with the hypothesis that the export and adenylation defects can be separated (11).

Nab2 appears to associate with most mRNAs before they are exported (17,22), and, although localized to the nucleus at steady-state, it shuttles between the nucleus and cytoplasm (10,12). Nab2 appears to become attached to the mRNP after splicing and during or immediately after polyadenylation (17,22) and influences the generation of export-competent mRNPs [15,17,reviewed by (1–8)]. Interestingly, mutation of the gene encoding the human Nab2 counterpart, *ZC3H14*, leads to an inherited form of intellectual disability (13,14), highlighting the importance of this protein in the brain of higher organisms.

**Figure 1.**
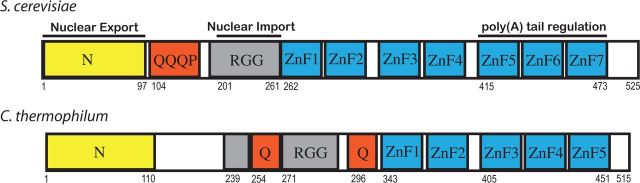
Domain architecture of *S. cerevisiae* and *C. thermophilum* Nab2. Both proteins contain an N-terminal domain based on a PWI fold (yellow) followed by an RGG domain (grey), a stretch of Gln-rich sequences (red) and finally a C-terminal domain (blue) that contains CCCH Zn-fingers (seven in *S. cerevisiae* and five in *C. thermophilum*). *Saccharomyces cerevisiae* fingers 5–7 are crucial for control of poly(A) tail length and BLAST analysis (Supplementary Figure S1) indicated that they are most similar to *C. thermophilum* fingers 3–5.

The *S. cerevisiae* Nab2 protein contains four domains (Figure 1): an N-terminal PWI-like domain that interacts with NPCs (23–25) followed by a Gln-rich linker; then an Arg-Gly (RGG) domain required for nuclear import (26); and finally a domain containing seven tandem CCCH Zn fingers that binds polyadenosine-RNA *in vitro*, contributes to poly(A) tail length control and is also a checkpoint for proper 3′ processing (13,27). The Zn finger domain is essential for Nab2 function and for its binding to poly(A) mRNA, albeit only fingers 5–7 are necessary and sufficient for high-affinity polyadenosine-RNA binding (27). Although Nab2 clearly binds to polyadenosine and mRNA poly(A) tails, this protein may also bind to other regions of the transcript in addition to the poly(A) tails and has been proposed to contribute to mRNP organization and compaction (22). Thus, yeast mRNPs isolated by TAP-tagged Nab2 pull-downs contain approximately nine Nab2 molecules per Kb (22), whereas poly(A) tails probably only bind two or three Nab2 molecules (28). Similarly, transcriptome-wide analysis of RNP composition (17) and chromatin immunoprecipitation (ChIP) studies (29) also indicate that Nab2 is bound throughout the body of mRNAs in addition to being concentrated at their 3′ poly(A) tail.

How Nab2 contributes to mRNP polyadenylation, assembly and surveillance is currently unclear. Although overexpression of *PAB1* [poly(A) binding protein 1] suppresses deletion of *nab2*, it does not correct the associated hyperadenylation (11). Moreover, addition of Nab2p and Pab1p did not lead to stimulation or inhibition of polyadenylation *in vitro*, whereas addition of Nab2p suppresses binding of Pab1p and the associated shortening of the poly(A) tail by the PAN (poly(A) nuclease) complex (28), suggesting that poly(A) tail length is influenced primarily by Nab2p and not Pab1p. Moreover, although both Pab1p and Nap2p are required to prevent polyadenylated transcripts being cleaved again by the cleavage and polyadenylation factor, addition of Nab2p prevents cleavage and polyadenylation factor adding further adenosines to mature polyadenylated transcripts, suggesting that Nab2 can more specifically prevent accessibility of the 3′ ends of mature tails to poly(A) polymerase, albeit both Nab2p and Pab1p inhibit poly(A) polymerase to comparable extents (28). Nab2 requires the Zn finger region plus an N-terminal moiety that includes the RGG box to perform its function in mRNA 3′-end formation (28). Deletion of the RGG domain (residues 201–264) showed some hyperadenylated transcripts and some of normal length, suggesting a partial involvement of the RGG domain (the N-terminal domain was still present). However, deletion of the RGG domain did not appear to decrease the affinity of Nab2 for poly(A). Deletion of the polyQ domain shows no influence on poly(A) tail length (28).

Because the function of Nab2 in polyadenylation relies critically on its recognizing poly(A) mRNA specifically, it is important to establish the structural basis for this molecular recognition. The solution structure of *S. cerevisiae* fingers 5–7 showed that these fingers form a novel coherent structure that binds to a total of eight adenosines, but it was not possible to establish precisely how these nucleotides were bound or to define how the Nab2 Zn fingers were able to distinguish adenosine from other nucleotides (15). The solution structure of Zn fingers 1–4 (30) indicates that both fingers 1 and 2 and fingers 3 and 4 form coherent pairs with an intervening helix. Fingers 1–4 bind ∼12 adenosines somewhat more weakly than fingers 5–7, but here too it was not possible to define the basis of molecular recognition (30). Here we describe the crystal structure of a complex between polyadenosine RNA and *Chaetomium thermophilum* Nab2 Zn fingers 3–5 that are homologous to *S. cerevisiae* fingers 5–7. As observed with the *S. cerevisiae* protein, (15) the three *C. thermophilum* Zn fingers have similar folds and associate into a single coherent structural unit. The crystal structure shows how the Nab2 Zn fingers are able to recognize adenosine specifically, primarily through the formation of H-bonds involving purine N6 and N7 nitrogens, which cannot be formed with other bases. Although eight adenosines are required to bridge *S. cerevisiae* Zn fingers 5–7 (15), the crystal structure indicates that probably only five of these bases are bound by the fingers, which would enable Nab2 to also recognize A-rich motifs in the transcript that contained key adenosines separated by spacer nucleotides in which the base was not crucial.

## MATERIALS AND METHODS

### Cloning, protein synthesis and purification

A synthetic gene corresponding to Nab2 residues 401–466 from *C. thermophilum* DSM 1495 (Accession Number EGS19143) and optimised for *Escherichia coli* expression was purchased from Genescript (Piscataway, USA) and cloned into the BamHI and NotI restriction sites into the pGEX-TEV vector (31). The construct was transformed into *E. coli* BL21-CodonPlus®(DE3)-RIL cells (Agilent). Cells were grown at 37°C to an OD_600_ of 0.5, when they were induced with 200 µM IPTG plus 50 µM ZnCl_2_ and grown at 20°C for further 16 h. Cells were harvested by centrifugation at 5000 *g* and resuspended in 50 mM Tris–HCl (pH 8.5), 200 mM NaCl, 300 µM ZnCl_2_, 3 mM DTT, 20% sucrose and stored at −20°C. Cells were lysed on ice by high-pressure cavitation at 10–15 Kpsi. Complete EDTA-free protease inhibitor mixture (Roche, Burgess Hill, UK), 1 µg/ml Deoxyribonuclease I (Sigma-Aldrich, St Louis, USA), 1 µg/ml Ribonuclease A (Sigma-Aldrich), 1 mM MgCl_2_ and 1 mM MnCl_2_, were added to the lysate, and the mixture was incubated at room temperature for 30 min. The lysate was clarified by centrifugation and bound to gluthathione Sepharose 4B resin (GE Healthcare, Amersham, UK) for 1 h at 4°C. The glutathione S-transferrase (GST)-tagged protein was eluted in 50 mM Tris–HCl (pH 8.5), 50 mM NaCl, 300 µM ZnCl_2_, 3 mM DTT, 20 mM reduced glutathione (Sigma-Aldrich). The GST tag was removed by incubating the protein overnight at 4°C with 100 µg of His-tobacco etch virus (TEV) protease [SV219V mutant (32)]. The Nab2 Zn finger protein was separated from the GST and TEV-protease by size exclusion chromatography using a HiLoad Superdex 75 26/60 column (GE Healthcare) equilibrated in 20 mM Tris–HCl (pH 8.5), 50 mM NaCl, 2 mM Mg acetate, 100 µM ZnCl_2_, 1 mM DTT.

### Crystallization and structure determination

The protein was concentrated to 60 mg/ml using Amicon centrifugal concentrators (Millipore, Billerica, USA). RNA with a sequence of AAAAAAAA (A_8_) was purchased from Integrated DNA Technologies (Leuven, Belgium) and dialysed against 20 mM Tris–HCl (pH 8.5), 50 mM NaCl, 2 mM Mg acetate, 100 µM ZnCl_2_, 1 mM DTT at 4°C overnight. RNA was added to the protein in a 1.2:1 molar ratio, and the mixture was diluted to a final protein concentration of 40 mg/ml. Protein–RNA crystals were obtained by hanging drop vapour diffusion in 20% PEG 4000, 300 mM MgCl_2_, 100 mM Tris–HCl (pH 8.5). Crystals in well solution supplemented with 20% glycerol were vitrified by plunging into liquid nitrogen. Crystallographic data were collected at beamline I02 at the Diamond Light Source, UK.

Initial phases were obtained using single anomalous dispersion (exploiting the anomalous signal of the six Zn atoms present in the asymmetric unit) using the AutoSol automated protocols in the *PHENIX* suite followed by AutoBuild (33). The resultant model was rebuilt manually and solvent flipping applied (34), which enhanced the clarity of the maps considerably. Iterative cycles of refinement were performed using *phenix.refine*, with local rebuilding in *COOT* (35) to give a final structure with an R_work_/R_free_ of 19.5/20.7% and excellent overall stereochemistry (Table 1) with a final MolProbity (36) score of 0.85 (100th percentile).

**Table 1.** Data collection and refinement statistics

Data collection	
Resolution range (Å)	45.47 - 2.15 (2.27 - 2.15)
Space group	*P 3_1_21*
Unit cell (Å)	90.9, 90.9, 54.1
Total reflections	129 094 (19 213)
Unique reflections	14 357 (2077)
Multiplicity	9.0 (9.3)
Completeness (%)	100 (100)
Mean I/σ(I)	12.4 (2.3)
Anomalous completeness (%)	100 (100)
Anomalous multiplicity	4.5 (4.6)
Wilson B-factor	46.74
R_pim_	0.042 (0.689)
Refinement	
R-factor/R_free_	0.1946/0.2066
Bonds RMS (Å)	0.003
Angles RMS (°)	0.87
Ramachandran favored (%)	99.2
Ramachandran outliers (%)	0
MolProbity score (percentile)	0.85 (100%)

Values for the highest-resolution shell are shown in parentheses.

### Physical biochemistry methods

Size exclusion chromatography–multi-angular light scattering used a Superdex 200 10/30 column coupled to a Wyatt Heleos II 18 angle light scattering instrument as described (37). Protein and RNA concentrations were determined from the excess differential refractive index (ΔRI), based on values of 0.186 for 1 mg/ml protein, 0.168 for 1 mg/ml RNA and 0.177 for 1 mg/ml protein–RNA complexes. The measurements were performed in 50 mM Tris (pH 8.5), 100 mM NaCl, 2 mM Mg Acetate, 100 µM ZnCl_2_ and 1 mM DTT. The molar mass determined as described (37). Isothermal calorimetry was performed in 50 mM Tris–HCl (pH 8.5), 50 mM NaCl, 10 µM ZnCl_2_ and 1 mM DTT as described using *S. cerevisiae* Zn fingers 5–7 (15). The stoichiometry of each measurement was normalised to 1 based on the RNA concentration, which was determined from the absorption at 260 nm.

## RESULTS AND DISCUSSION

### Crystal structure of *C. thermophilum* Zn fingers 3–5

**Figure 2.**
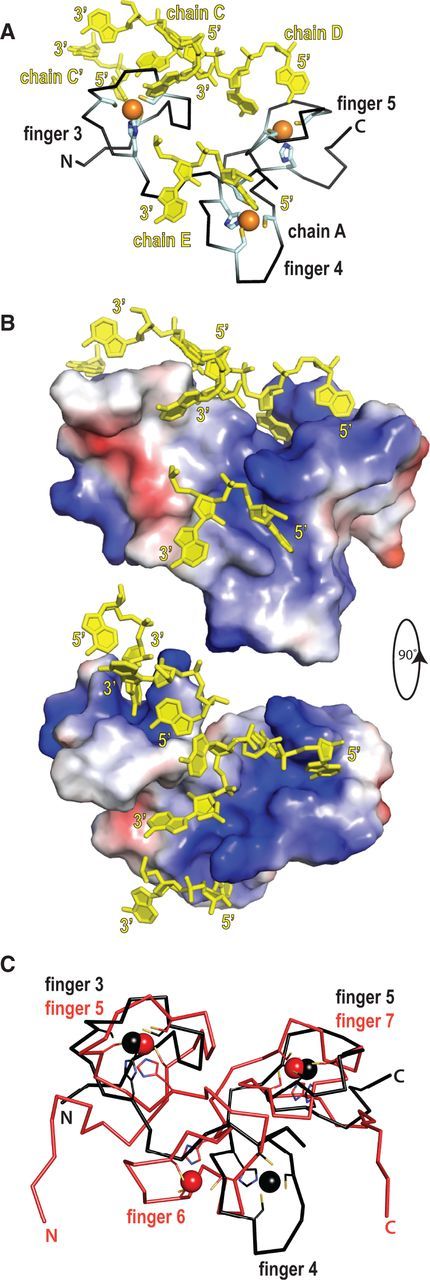
Structure of Nab2 Zn fingers 3–5 in complex with poly(A) RNA. The X-ray structure was determined from *C. thermophilum* Nab2 Zn fingers 3–5. (**A**) The purine bases of the polyadenosine RNA (yellow) bind to surface grooves on the protein (black), whereas the RNA ribose-phosphate backbone is oriented away from the protein and lies over its surface. Zn (orange balls) is coordinated from cysteine and histidine residues (cyan stick representation). (**B**) The calculated electrostatic surface potential (± 50 kT/e) of the fingers shows the positive charged environment (blue) of the RNA (yellow) binding site of the Nab2 Zn fingers. (**C**) Superposition of the C-α traces of the crystal structure of Nab2 Zn fingers 3–5 from *C. thermophilum* (black) and the NMR structure of Nab2 Zn fingers 5–7 from *S. cerevisiae* (red), which was solved in the apo-state. The Zn fingers of both proteins have the same overall fold. However, whereas the terminal Zn fingers superimpose well, the middle Zn finger is rotated by ∼53° between the *C. thermophilum* and *S. cerevisiae* proteins.

**Figure 3.**
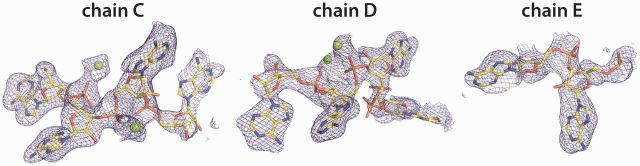
*2Fo-Fc* electron density for each polyadenine RNA chain in the *C. thermophilum* Nab2 crystal structure and its associated Mg^2+^ ions (green spheres). Both the purine bases and the RNA ribose-phosphate backbone could be fitted unambiguously into the electron density. For clarity, the surrounding protein and solvent atoms have been omitted, but are shown, together with the network of H-bonds that determines binding specificity, in Figure 4.

Although attempts to obtain crystals of *S. cerevisiae* Nab2 Zn fingers 5–7 complexed with polyadenosine RNA were unsuccessful, it was possible to obtain crystals using a construct obtained from the thermophilic yeast *C. thermophilum*. Sequence analysis using BLAST indicated that Zn fingers 3–5 of *C. thermophilum* Nab2 showed the highest level of homology to the fingers 5–7 of *S. cerevisiae* Nab2 (Supplementary Figure S1). The DNA corresponding to fingers 3–5 (residues 401–466) was synthesised and the protein expressed in *E. coli*. The resultant protein formed a complex with single-stranded polyadenosine RNA containing eight nucleotides that multi-angle light scattering indicated contained one protein chain and one RNA chain. Thus, the apparent M_r_ of Zn fingers 3–5 alone was 8.2 kDa (theoretical 7.7 kDa), that of A_8_ RNA 3.4 kDa (theoretical 2.6 kDa) and that of fingers 3–5 complexed with A_8_ RNA 10.6 kDa (theoretical 10.2 kDa for a 1:1 complex). This material was used to generate crystals that had *P3_1_2_1_* symmetry with two protein:RNA complexes in the asymmetric unit. The structure was solved by phasing with the anomalous signal from the six protein-bound Zn atoms followed by iterative rounds of model building and refinement. The final 2.15 Å resolution structural model had an R-factor of 19.5% (R_free_ 20.7%) and excellent geometry (Table 1), with a final MolProbity (36) score of 0.85 (100th percentile). The final model (Figure 2) contained two copies of Nab2 Zn fingers 3–5 (chains A and B) with three Zn atoms each, two acetate molecules, four magnesium ions and 25 water molecules. Additionally, three poly(A) RNA chains were unambiguously placed into the electron density (Figures 2 and 3), two of which contained four nucleotides (chains C and D) and the third contained two nucleotides (chain E). All nucleotides placed into the model showed substantially higher B-factors than the protein chains. Each RNA chain bound to two different protein chains (Supplementary Figure S2), probably as a result of a form of domain swapping induced by the packing of the molecules into a crystalline lattice. A similar domain swapping was observed for the interaction between MLB1 and RNA (38). Generally, the RNA chains were defined most clearly when they were in direct contact with the protein (Supplementary Figure S3). Although some less well-defined electron density was observed linking the nucleotide chains, this was not sufficiently clear to enable a reliable model to be built in these regions. The electron density of the adenosine bases bound to each Zn finger and their associated ribose and phosphate was unambiguous and well-defined (Figure 3), which enabled the structural basis of molecular recognition of polyadenosine RNA by each Nab2 Zn finger motif to be established unambiguously.

### Overview of the structure

The final model contained two protein chains with three Zn fingers each and three separate RNA chains with each one being in contact with more than one protein chain. Although the three Zn fingers lacked secondary structural elements, they were packed into a single coherent structure that closely resembled that seen with *S. cerevisiae* fingers 5–7 (15), albeit superimposition of both structures using their terminal Zn fingers (Figure 2C) indicated that the middle Zn finger (Zn finger 6 in *S. cerevisiae* and Zn finger 4 in *C. thermophilum*) had rotated by ∼53° relative to the terminal fingers. However, the structure of individual fingers was strongly conserved, and, for example, superimposition of the corresponding single Zn fingers from each protein had RMSD values ranging between 1.2 and 1.8 Å. The RNA backbone was arranged on the protein surface, whereas the purine bases were buried into characteristic pockets formed in the Zn fingers. Overall, the density of protein chain A together with its bound nucleotides was defined considerably more clearly than chain B, where, although all the major structural features were conserved, the B-factors were higher and, in some parts of the chain, the electron density was less well-defined than for chain A. Consequently, chain A was generally used for detailed analysis of the protein structure and its interactions with RNA.

### Protein–RNA interaction

Poly(A) RNA binding to Nab2 Zn fingers involved mainly its purine bases, which insert into specific binding pockets on each finger. The primary contacts between the RNA and the protein were formed by the adenine bases, whereas the ribose and phosphate backbone was oriented towards the solvent-exposed surface and made only marginal contributions to RNA binding. All four Mg^2+^ ions placed into the electron density were coordinated to oxygens of the RNA backbone phosphates and therefore contribute to neutralizing its negative charge.

**Figure 4.**
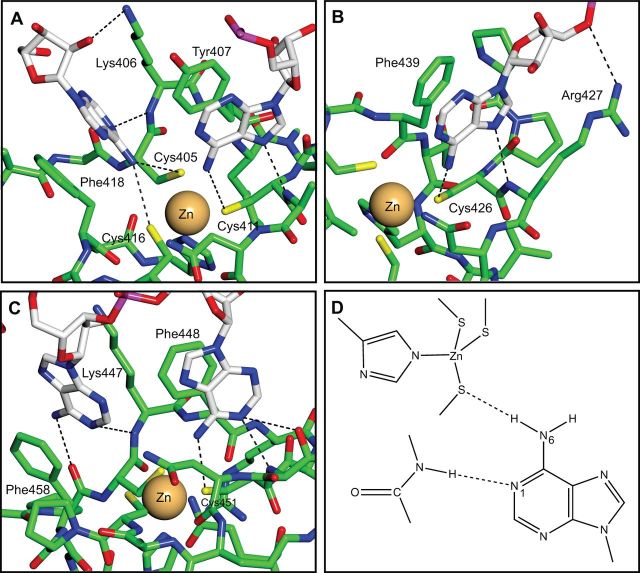
H-bonding pattern that results in *C. thermophilum* Nab2 fingers 3 (**A**), 4 (**B**) and 5 (**C**) recognizing adenosine selectively. In fingers 3 and 5, two adenosines are bound, whereas finger 4 binds only a single adenosine. In each case, the adenine purine ring is bound to a groove on the surface of the finger and is stacked against an aromatic protein side chain (Tyr407 and Phe418 in finger 3; Phe439 in finger 4; and Phe448 and Phe458 in finger 5) and frequently a basic side chain (such as Lys406 or Lys447). The ribose-phosphate backbone of the RNA lies on the surface of the finger and forms relative few specific interactions with the protein. Specificity for adenosine binding is provided by a network of H-bonds, primarily between the purine ring and the protein. Protein carbons are green and nucleotide carbons are white. (**D**) Schematic illustration of the most common pattern of H-bonds, involving those between the adenine N6 and the SG atom of cysteines bound to Zn; and between the adenine N1 and an adjacent peptide NH.

Nab2 protein chain A interacted with five nucleotides overall, with Zn fingers 3 and 5 each binding two adenosines and Zn finger 4 interacting with one (Figures 2 and 4). In each binding pocket, the purine base stacked against an aromatic side chain with the hydrophobic region of commonly a lysine or arginine residue masking its other face. There also often appeared to be putative π interactions with these basic residues. Thus, the position of Lys406 and Lys447 appeared to be consistent with a cation–π interaction (Figure 4A and B). However, although the position of Arg427 in the A-chain did not match the criteria for a cation–π interaction, this appeared to be due to its forming a putative H-bond with the phosphate of A2 of chain E in the crystal lattice.

The interaction of chain A with RNA buried 1893 Å^2^ of surface area that was primarily the result of the purine rings stacking in surface grooves on the Zn fingers. In addition, each base formed key H-bonds between the N6 and N1 or N7 atoms of the purine ring and the protein. Thus, finger 3 bound nucleotides A1 and A2 of chain C, with A1 stacked between Phe418 and Lys406 and A2 stacked against Tyr407. Putative H-bonds were formed between nitrogen N6 of both adenines and the SG atom of a Zn coordinating cysteine (A1 to Cys405 and Cys416; A2 to Cys411). Base A1 was oriented with its Watson–Crick edge towards the Zn coordination site, enabling its N1 nitrogen to form an additional putative H-bond with the main-chain amide of Lys406, whereas the A2 adenine of chain C presented its Hoogsten edge towards the Zn so that the amide N-H of Ala412 was in an optimal orientation for forming a putative H-bond with the purine N7. These bases were also linked by solvent-mediated H-bonds (Supplementary Figure S3). Water S15 interacted with N6 in each base and water S7 with N1 and N7 in A2 and A1, respectively. Zn finger 4 recognized only a single adenosine, A2 of chain E, that was oriented such that its Hoogsten edge was facing the CCCH-Zn cluster. The base of A2 was stacked between Arg427 and Phe439 and H-bonded with N1, N6 and N7 to water S18 and S19, the SG sulphur of Cys426, and the main chain amide nitrogen of Arg427, respectively. Zn finger 5 bound two nucleotides in chain D: adenine A3 was stacked between Phe458 and Lys447 and A4 stacked against Phe448. Both were oriented with the Watson–Crick edge facing the Zn. However, whereas A3 had putative H-bonds via N6 and N1 to the peptide carbonyl of Pro445 and main chain amide of Lys447, respectively, A4 had putative H-bonds via N6 and N1 to the SG of Zn coordinating Cys451 and the main chain amide of Thr452, respectively.

In summary, all bases were bound to the Zn fingers via stacking interactions with an aromatic side chain, and, except of adenine A3 of chain D, they all formed putative H-bonds between the purine N6 and a Zn coordinating cysteine residue and N1 or N7 with the subsequent main chain nitrogen (Figure 4D). This hydrogen-bonding pattern was observed irrespective of the orientation of the bases, i.e. facing with their Hoogsten or Watson–Crick edge into the binding pocket of the protein.

### Molecular recognition of adenosine

*Saccharomyces cerevisiae* Nab2 Zn fingers 5–7 bind poly(A) RNA with ∼100 nM affinity (15), whereas no binding was detected to poly(C), poly(G), poly(U) RNA (Supplementary Figure S4). As illustrated in Figure 4D, molecular recognition of polyadenosine RNA by *C. thermophilum* Nab2 Zn fingers is based on the formation of H-bonds by the N6 nitrogen of the purine base augmented by π interactions with adjacent aromatic residue and, in four cases out of five, a positively charged side chain. In fingers 3 and 4, N6 forms H-bonds with the SG of Zn coordinating cysteines and N7 forms H-bonds with an adjacent main-chain carbonyl oxygen. Adenine N6 is a H-bond donor, whereas in guanine this position is occupied by the O6 atom that is a H-bond acceptor that cannot bond to the SG of the cysteine because its hydrogen is lost when bound to Zn^2+^. The interaction between finger 5 and the adenines is similar to that observed with fingers 3 and 4, albeit with some differences in detail. Thus, although N6 of adenine A4 of chain D forms a putative H-bond to the SG of Cys451, N7 does not approach the Nab2 main chain and instead N1 forms H-bonds with the main chain amide of Thr452. Adenine A3 of chain D instead forms an H-bond to the peptide carbonyl of Pro445 with N7. Although a cytosine pyrimidine N4 could in principle form an H-bond to the SG of a cysteine residue, it would be unable to form a second H-bond because it lacks a corresponding N7 and so would be expected to bind more weakly, whereas uridine would be analogous to guanine and so unable to participate in the H-bond network. Consequently, only adenosine is able to bind with high affinity to the Nab2 Zn fingers.

Although in solution, *C. thermophilium* Zn fingers 3–5 formed a 1:1 complex with A_8_ RNA, in the crystals domain swapping resulted in each protein chain binding to three different RNA chains (Supplementary Figure S2). This sort of domain swapping is seen in other Zn finger crystals (38) and probably results from the binding of each individual adenine to a finger being comparatively weak so that the RNA chains are able to rearrange in the crystal. Consequently, although the crystal structure established the molecular basis for the recognition of adenine in preference to other nucleotides, it does not establish the precise path followed by A_8_ on the surface of a single protein chain in solution and raises the question of whether it would be possible for a single A_8_ chain to bind to all five sites identified in the crystal. We therefore investigated whether it was possible to build models consistent with this idea and found that this could be accomplished relatively easily. Supplementary Figure S5 shows one of many possible models that can be constructed in which a single protein chain containing fingers 3–5 binds five adenines in a single A_8_ chain that has normal bond lengths (RMSD 0.005 Å) and angles (RMSD 0.78°) and in which the five adenine bases arranged to have the most common interaction geometry identified in the crystals (Figure 4D). Although this model shows that such an interaction can occur, further work will be required to establish experimentally the precise path followed by the A_8_ chain on *C. thermophilium* Zn fingers 3–5.

### Comparison with other RNA-binding Zn finger domains

The molecular recognition of adenosine by Nab2 Zn fingers differed considerably from the pattern of interactions observed in the interactions between TIS11d (39) and MBNL1 (38). Thus, although the adenines in Nab2 are stacked against an aromatic residue and form putative π interactions with a basic residue that is frequently observed when single-stranded RNAs bind to proteins (40–42), the pattern of H-bonds that generated the specificity of the interaction (Figure 4D) was different to those observed previously. Thus, TIS11d binds to UAUU RNA sequence motifs in which the N6 and N7 of the adenine purine form H-bonds to main-chain carbonyls or amides and do not interact with the SGs from the Zn-coordinating cysteines (39). MBNL1 binds to CG steps that are dominated by a network of H-bonds formed primarily with the protein main chain (38). Although the guanines form H-bonds with the Zn coordinating cysteine SGs, these involve purine N1 and N2, with O6 H-bonded to a main-chain carbonyl, whereas the cytosine pyrimidines are H-bonded almost exclusively to the protein main-chain and do not form any H-bonds to the Zn coordinating cysteine SGs.

### Conservation of key binding residues between Nab2 Zn fingers

**Figure 5.**
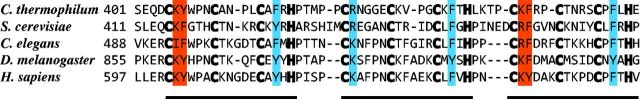
Multiple sequence alignment of Nab2 proteins from different species shows strong conservation of the residues involved in binding adenosine specifically in the crystal structure of *C. thermophilum* fingers 3–5. The Zn-binding cysteine and histidine residues are shown in bold. The basic and aromatic residues (red) that follow the first cysteine and the aromatic residue (cyan) that follows the third Cys of the CCCH motif are strongly conserved. Most of the residues conserved between species are involved in either Zn-coordination or RNA binding.

Adenosine recognition in Nab2 followed a general pattern of N6 binding to the first or second cysteine in the CCCH Zn finger with N1 or N7 binding to the following main chain amine NH (Figure 4). The highly conserved lysine and arginine residues that follow the first cysteine residue in each finger (Lys406, Arg427, Arg447) function in base stacking, contributing to neutralizing the negative charge of the RNA backbone and providing a main chain amine that contributes to the recognition of the adenine bases. The aromatic residues Tyr407, Phe418, Phe439, Phe448 and Phe485 (that are all involved in base stacking) were always located at the second position after the first or third cysteine of the finger, except for ZnF4, which has only an aromatic residue after the third cysteine. All these aromatic residues were highly conserved between species (Figure 5). The adenine-binding residues identified in the crystal structure are consistent with mutagenesis results from *S. cerevisiae* Nab2. Mutations of the key aromatic residues and basic residues all result in decreased affinity for A8 RNA and production of longer poly(A) tails (15).

**Figure 6.**
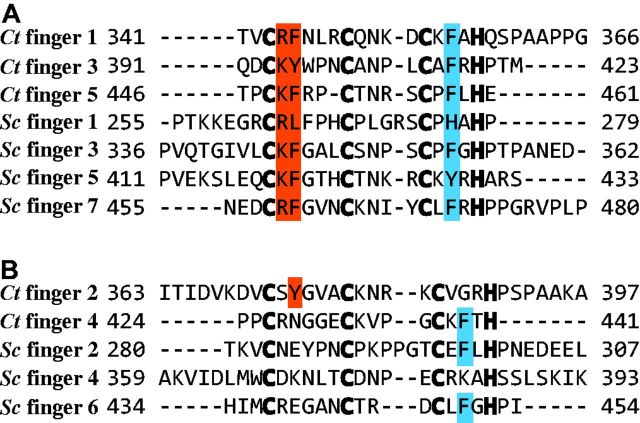
Sequence alignment of individual Nab2 Zn fingers from *S. cerevisiae* and *C. thermophilum*. The Zn-binding cysteine and histidine residues are shown in bold. (**A**) All of the odd-numbered Zn fingers contain the highly conserved basic and aromatic residue (red box) after the first cysteine of the finger and the highly conserved aromatic residue after the third cysteine that are crucial for binding adenosine specifically. In *C. thermophilum*, fingers 3 and 5 bind two adenosines, and the pattern of conservation indicates that this would also be the case for the other fingers in this group. (**B**) The remaining Zn fingers appear to retain only one of the two adenosine-binding motifs and, by analogy to *C. thermophilum* finger 4, are thought to bind only a single base.

Sequence analysis (Figure 6) together with the structures of *S. cerevisiae* fingers 1–4 (30) and 5–7 (15) and *C. thermophilum* fingers 3–5 (Figure 2) indicates that the Zn fingers in both *C. thermophilum* and *S. cerevisiae* Nab2 clearly fall into two groups: fingers that contain aromatic residues in the second position after the first and third Cys of the CCCH motif and which would bind two adenosines (such as *C. thermophilum* fingers 3 and 5 or *S. cerevisiae* fingers 5 and 7) and those that have an aromatic residue at only one of these positions in the finger and bind only a single adenosine (such as *C. thermophilum* finger 4 or *S. cerevisiae* finger 6). In *S. cerevisiae* Nab2, sequence analysis (Figure 6) indicates that fingers 3, 5 and 7 would bind two adenosines, whereas fingers 2, 4 and 6 would bind only one. Although *S. cerevisiae* finger 1 does not fit the consensus precisely, it does have Leu in position 1 and His in position 14, which could enable it to also bind two adenosines. An important implication of this observation is that, although the Nab2 Zn fingers show sequence motifs consistent with their binding adenosine exclusively, between the bound adenosines there are probably spacer nucleotides in which the identity of the base is not crucial. Thus, *S. cerevisiae* Nab2 fingers 5–7 bind to a total of eight nucleotides (15) but, by analogy with the *C. thermophilum* structure, only five of these nucleotides are probably recognized by the protein (two by fingers 5 and 7 and one by finger 6). Thus, although Nab2 will therefore clearly bind to polyadenosine RNA, it could also bind to A-rich sequences in which the adenosines are spaced at appropriate intervals, although avidity considerations would result in polyadenosine sequences, especially 3′ poly(A) tails, binding more strongly that the appropriate A-rich sequences. Consequently, poly(A) sequences would be expected to be isolated preferentially by any selection procedure based on affinity and so would be consistent with the finding that co-immunoprecipitatation analysis for Nab2 identified a 12-nt motif with the sequence AAAAAAAAAAAG (43).

In addition to their function in binding to poly(A) tails and regulating their length, Nab2 has also been proposed to function in mRNA nuclear export as well as compaction and organization of mature mRNPs (16,22). Pull-down experiments with TAP-tagged Nab2 indicate that ∼12 Nab2 molecules are bound per Kb of mRNA (22), whereas only two or three Nab2s would be expected to bind to the ∼70 nt poly(A) tail (28) present on *S. cerevisiae* transcripts, suggesting that Nab2 may also bind to regions of the transcript outside the poly(A) tail. Similarly, transcriptome-wide analysis of RNP composition (17) and ChIP studies (29) also indicate that Nab2 is bound throughout the body of mRNAs in addition to being concentrated at their 3′ poly(A) tail. It has been proposed that additional Nab2 molecules bound to the bulk of the transcript are important for mRNP organization and compaction as well as surveillance (17,22). The ability of Nab2 to bind to sequences in which key adenosines were located with the appropriate spacing could enable Nab2 to bind to regions of many transcripts located outside their poly(A) tails. In principle, these interactions could involve either spacer residues filling the gaps between bound adenosines in a continuous sequence or, alternatively, could involve binding to separated adenosine-rich clusters to enable tethering of highly remote regions and thereby contribute to transcript compaction. The way in which the RNA chains swap between different protein chains in the crystal (Supplementary Figure S2) could possibly indicate how the Nab2 fingers could bind to remote A-rich clusters, but further experimental work will be required to evaluate these possibilities.

In summary, the crystal structure of *C. thermophilum* Nab2 Zn fingers 3–5 demonstrates the molecular basis for their selectivity in binding poly(A) RNA. Although in common with other CCCH Zn fingers, the purine bases are intercalated between an aromatic side chain and frequently a basic residue, in Nab2, sequence specificity in recognition is achieved by a specific network of H-bonds between the protein and N7 and N6 of the adenine purine. Moreover, the pattern of binding indicates that, whereas Nab2 Zn fingers 1, 3, 5 and 7 appear to bind two adenosines, fingers 2, 4 and 6 appear to bind only a single nucleotide and provides an explanation for Nab2 binding to A-rich sequences in which specific key adenosines can be separated by spacers containing any nucleotide. This, in turn, could account for Nab2 binding to regions in the bulk of the transcript and contributing to its organization and compaction (22) in addition to its function in regulating poly(A) tail length (11,13,15,27,28).

## ACCESSION NUMBERS

Coordinates and structure factors have been deposited at the RCSB PDB with accession code 4LJ0.

## SUPPLEMENTARY DATA

Supplementary Data are available at NAR Online.

## FUNDING

Medical Research Council (MRC) grant [105178939]; Wellcome Trust Programme grant (to M.S.). Funding for open access: MRC.

*Conflict of interest statement*. None declared.

## Supplementary Material

Supplementary Data
