# Supplementary material for: Structural basis for the molecular recognition of polyadenosine RNA by Nab2 Zn fingers
Source: Nucleic Acids Res. 2013 Sep 25;42(1):672–80. doi: 10.1093/nar/gkt876 (PMC3874189; doi:10.1093/nar/gkt876)
Supplement: Supplementary Data [file supp_42_1_672__index.html]

Structural basis for the molecular recognition of polyadenosine RNA by Nab2 Zn fingers — Structural basis for the molecular recognition of polyadenosine RNA by Nab2 Zn fingers — Supplementary Data 

# Structural basis for the molecular recognition of polyadenosine RNA by Nab2 Zn fingers

## Supplementary Data

files

**Files in this Data Supplement:**

- Supplementary Data - pdf file
