# Supplementary material for: Structural basis for the molecular recognition of polyadenosine RNA by Nab2 Zn fingers
Source: Nucleic Acids Res. 2013 Sep 25;42(1):672–80. doi: 10.1093/nar/gkt876 (PMC3874189; doi:10.1093/nar/gkt876)
Supplement: Supplementary Data [file supp_gkt876_nar-02052-h-2013-File008.pdf]

SUPPLEMENTAL INFORMATION

Structural Basis for the Molecular Recognition of Polyadenosine RNA by Nab2 Zn Fingers

Sonja I. Kuhlmann, Eugene Valkov and Murray Stewart

MRC Laboratory of Molecular Biology, Francis Crick Avenue, Cambridge Biomedical Campus, Cambridge CB2 0QH, UK.

A

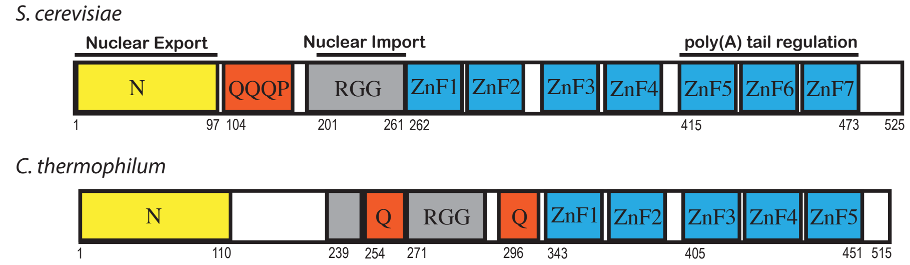

B

```
S. cerevisiae     MSQE-----QYTENLKVIVAEKLAGI--PNFNEDIKYVAEYIVLLIVNGGTVESVVDELA 53
C. thermophilum   MSVEIALGTPLADALNTAIQGKIAELGWAGPGSEVSAMSEYFVLMLANGKTEAEIVQEIQ 60
                  ** *        :: *:. :  *:* :  .. ..::. ::**:**::.** *  .:*:*:

S. cerevisiae     S-LF----DSVSRDTLANVVQTAFFALEALQ-QGESAENIVSKIRMMNAQSL-GQSDIAQ 106
C. thermophilum   GDLLGLGPEDETAPKFARWLSEQVAALSSQSGNGASAAPSGDAMDGIRDESMTGPSDVNM 120
                  . *:    :. :  .:*. :.  . **.: . :* **    . :  :. :*: * **:

S. cerevisiae     QQQQQQQQQQPDIAQQQPQQQPQQQPQQQPQQQPQQQPQQQPQQQPQQQPQLQPLQPQLG 166
C. thermophilum   DTSTDMPATGLN-APTGPKAMRNGNNMMRGSREKRLMGQISRAMDRSQDSVLHRVRTQSG 179
                  : . :       : *   *:   : :   : .:: :    * .    : .*:. *: ::.* *

S. cerevisiae     TQNAMQTDAPATPSP-ISAFSGVVNAAAPPQFAPVDNSQRFTQRGGGAVGKNRRGGRGGN 225
C. thermophilum   NE-RISRGPPAGPRLGVGRQPRTTNTRMASIAAGMANMSGMPHPGG-PMPMNPMNG---- 233
                  .:  :. ..** *   :.  . ..*:  ..  * : * . :.: ** .:  *   .*

S. cerevisiae     RGGRNNNSTRFNPLAKALGMAGESNMNFTPTKKEGRCRLFPHCP-LG-RSCPHAHPTKVC 283
C. thermophilum   MPPMPPNGTPYVP-PELYAMMEQQNLMLQQMQQQLQMQQASMRGGFGGRGGRGGGFRRGG 292
                        *.* : * .:  .*  :.*: :   ::: : :  .     :* *.    .    :

S. cerevisiae     NEYPNCPKPPGTCEFLHPNEDEELMKEMERTR-EEFQKRKADLLAAKRKPVQTGIVLCKF 342
C. thermophilum   GHFQQQHN--G--SHAHPQQQQSQQAGSEGTQSEGASQGDAEMAGAKPNPEET---VCRF 345
                  ..: :  :  *  .. **::::.     * *: *  .: .*:: .** :* :*   :*:*

S. cerevisiae     GALCSNPSCPFGHPTPANEDAKVIDLM-WCDKNLTCDNPECRKAHSSLSKIKEVKPISQK 401
C. thermophilum   NLRCQNKDCKFAHQSPAAPPGITIDVKDVCSYGVACKNRKCVGRH-----------PSPAA 395
                  .  *.* .* *.* :**    . .**:   *. .::*.* :*    *          * .

S. cerevisiae     KAAPPPVEKSLEQCKFGTHCTNKRCKYRHARSHIMCREGANCTRIDCLFGHPINEDCRFG 461
C. thermophilum   KAA----HQSEQDCKYWPNCANPLCAFRHP-TMPPCRNGGECKVPGCKFTH-LKTPCKFR 449
                  ***    .:* ::**: .:*:*  * :**. :   **:*.:*.  .* * * ::  *:*

S. cerevisiae     VNCKNIYCLFRHPPGR--VLPEKKGAAPNSNVPTNERPFALPENAIIENAPPQTSFTHQE 519
C. thermophilum   P-CTNRSCPFLHEEGQRGTFQDKVWTADGAKEHVSERKFAN-DTGNEELVLPGSGQQEEN 507
                    *.*  * * *  *:  .: :*  :* .::  ..** **  :..  * . * :.  .::

S. cerevisiae     Q-DTEMN- 525
C. thermophilum   QTETEVIM 515
                  * :**:
```

Supplementary Figure S1: Domain architecture (A) and sequence alignment (B) of *S. cerevisiae* and *C. thermophilum* Nab2. Both proteins contain an N-terminal domain based on a PWI fold (yellow) followed by an RGG domain (gray), a stretch of Gln-rich sequences (red), and finally a C-terminal domain (blue) that contains CCCH Zn-fingers (seven in *S. cerevisiae* and five in *C. thermophilum*). *S. cerevisiae* fingers 5-7 are crucial for control of poly(A) tail length and BLAST analysis indicated that they are most similar to *C. thermophilum* fingers 3-5.

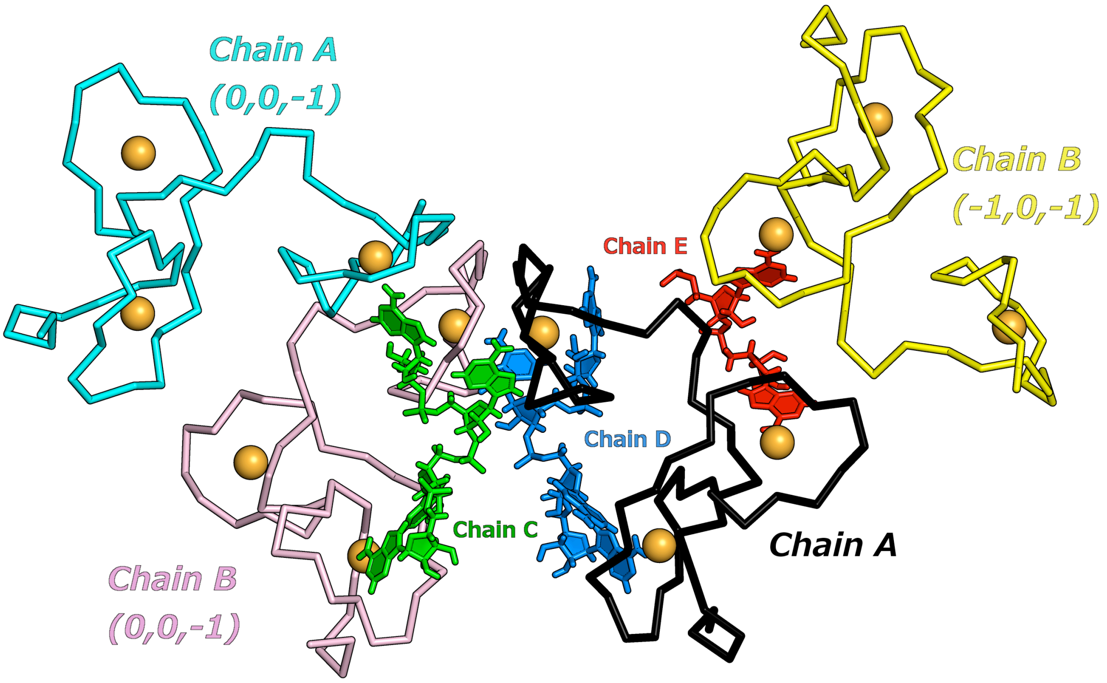

Supplementary Figure S2. Domain swapping in the crystal lattice resulted in the three different RNA chains binding to different pairs of protein chains. RNA chain C (green) is bound to protein chain A (black) and to the (0,0,-1) symmetry mate of protein chain A (cyan); RNA chain D (blue) is bound to protein chains A (black) and to the (0,0,-1) symmetry mate of chain B (pink); and RNA chain E (red) is bound to chain A (black) and the (-1,0,-1) symmetry mate of chain B (yellow).

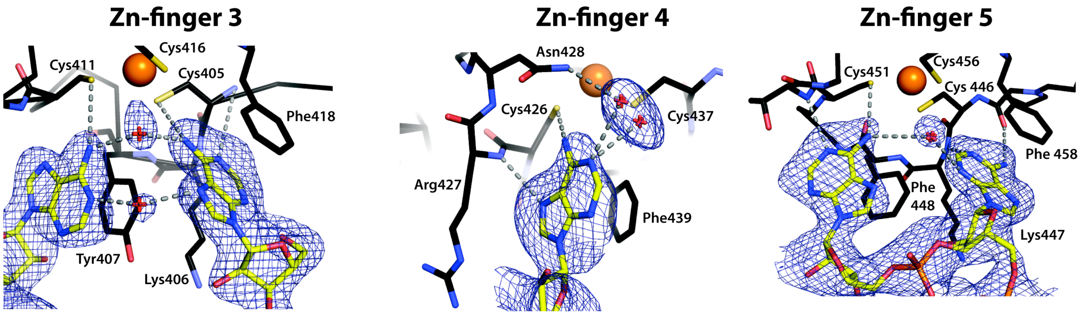

Supplementary Figure S3: H-bonding networks in the adenine-specific RNA-binding pockets of the three *C. thermophilum* Zn fingers. The RNA adenine bases (yellow) are stacked between lysine or arginine and aromatic residues in grooves on each Zn finger (black) and form H-bonds with Cys SG atoms, peptide backbone groups and several waters (red crosses). The 2Fo-Fc omit map carved around the RNA illustrates the reliability of these assignments.

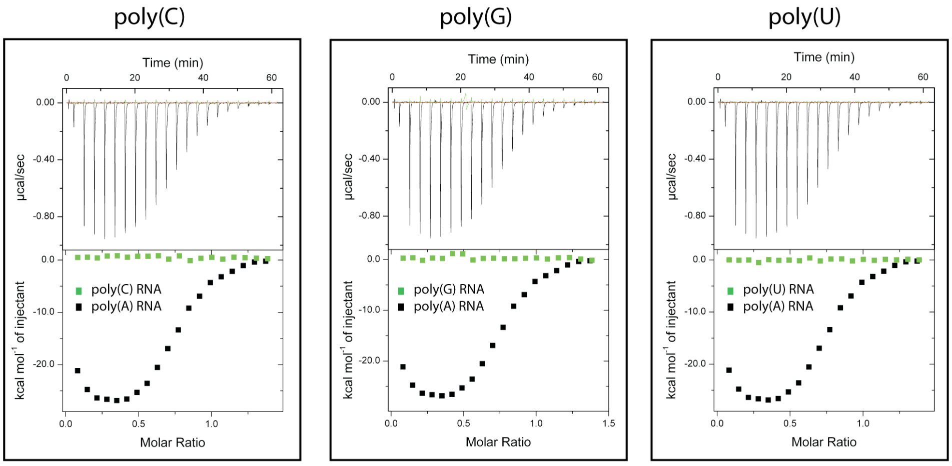

Supplementary Figure S4: Selectivity of Nab2 Zn fingers for polyadenosine. Isothermal calorometric titrations comparing the binding of poly(C), poly(G), or poly(U), with poly(A) RNA to Zn fingers 5-7 from *S. cerevisiae* Nab2. Each polyribonucleotide contained eight bases. Whereas *S. cerevisiae* Zn fingers 5-7 showed tight binding with a Kd of 0.5 µM, there was no detectable binding to poly(C), poly(G) or poly(U) RNA, indicating that these had substantially lower affinity for the Nab2 Zn fingers.

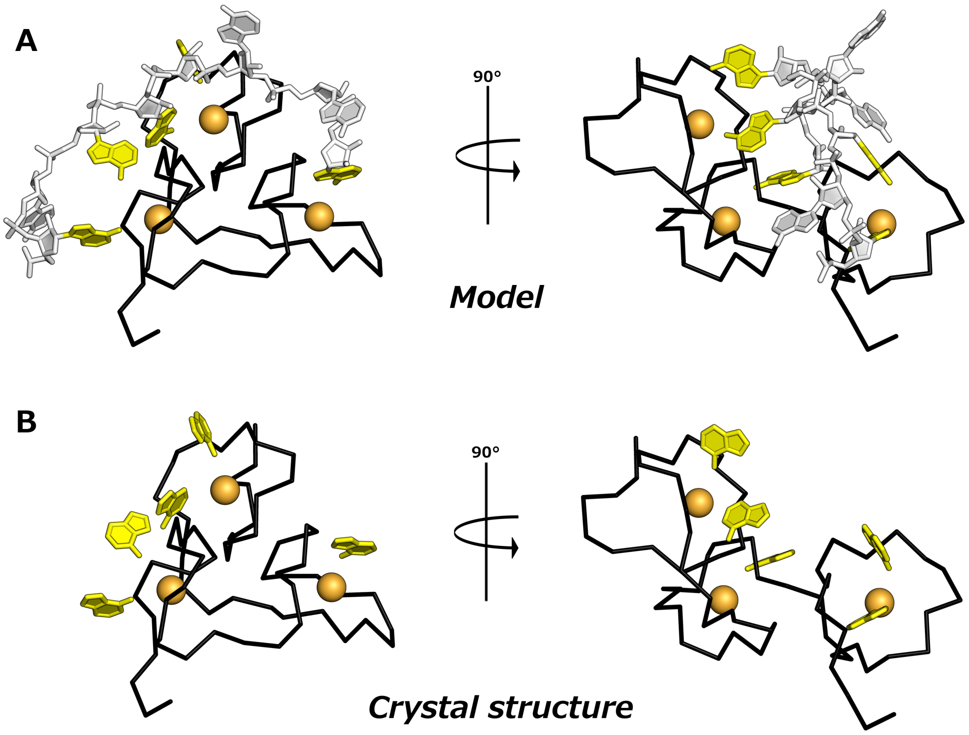

Supplementary Figure S5. A model illustrating how a single A8 RNA chain could bind to *C.thermophilum* Nab2 Zn fingers 3-5. A model A_8 RNA bound to chain A of the crystal structure was constructed using COOT (35). The structure of the crystal A chain was unaltered and the bond length (0.005 Å) and bond angle (0.74˚) rmsds of the RNA were within the normal range. In this model (A), five adenine rings are bound in the same positions as the adenines seen in the crystal structure (B) and with the orientation depicted in Figure 4D. The bound adenines are shown in yellow, whereas unbound adenines and the RNA backbone are white. Although this model demonstrates that it is possible for a single A_8 RNA to bind to Zn fingers 3-5, we stress that this is one of many possibilities and further work will be needed to define the precise path followed by this chain on the surface of the fingers in the complex.
